# Personal

**Published:** 1914-11

**Authors:** 


### PERSONAL.

---

Announcement of removal, travel, and other matters of interest are requested. Please report errors in the listing of any physician in the State and other directories, that we may co-operate with the State Society in securing a correct list.

---

Dr. D. T. Quigley announces his removal from North Platte, Nebraska to Omaha. He will devote his attention to surgery and radium therapy.

---

The automobile of Dr. Wm. H. Marcy was stolen from in front of a house on Bidwell Parkway, Buffalo, where he was calling, late Saturday evening, October 17. The car was found in a vacant lot in Kensington, Sunday. The tachymeter had been stolen and the car was otherwise damaged but not seriously. The doctor's medical satchel had been rummaged but nothing taken.

---

Two tires, with cases, were cut from the holder of the vehicle of Dr. A. L. Benedict, between 6 and 7.30 of the same evening, at the corner of Elmwood Avenue and Summer Street, presumably by men in another machine.

---

Dr. Lloyd Mottram has moved from Elmira to Mayville, N. Y. (October 10th)

Dr. Wm. S. Cain has been appointed health officer of Elmira.

---

Dr. S. L. Harrison of New York City has associated himself with Dr. Sherman Vorhees at Elmira. Practice limited to Eye and Ear.

---

Dr. D. E. Wheeler of Buffalo has sailed for France as a volunteer surgeon under the fund for transportation donated by the Duchess de Talleyrand.

---

Dr. J. J. Twohey of Buffalo returned in September from a two months' trip to Europe.

---

The office of Dr. Harry M. Weed of Buffalo was entered by a thief on the night of September 4-5, and rummaged but nothing taken.

---

Dr. Clara A. March of Buffalo has moved her office to 297 Delaware Avenue. Practice, as heretofore, limited to diseases of the eye.

---

Our Associate Editor, Hon. Henry W. Hill, LL.D. has been re-elected President of the International Water-Ways Commission.

---

Dr. Robert Hinds of Buffalo has been assigned to the base unit of the Red Cross in England.

---

Dr. A. A. Thibaudeau of Buffalo announces the opening of a Laboratory for Clinical Diagnosis at 23 Irving Place.

---

Dr. V. A. Wickens of Rochester has moved to 567 W. Main Street.

---

Dr. W. B. Cochrane of Rochester has moved to 571 Park Avenue.

---

Dr. Carl G. Leo-Wolf of Buffalo, late of Niagara Falls, has been elected President of the 8th District Branch of the Medical Society of the State of N. Y. Dr. A. T. Lytle, of Buffalo and Dr. J. T. Torre of Olean, Vice-presidents; Dr. E. A. Sharpe of Buffalo, Secretary; Dr. C. A. Wall of Buffalo, Treasurer.

---

Dr. John F. Beiermeister of Rochester, has moved to 353 University Avenue.

---

Dr. L. G. Hanley of Buffalo spent three weeks in September at his summer home in Woodmont, Conn.

Dr. George R. Critchlow of Buffalo has moved to 647 Lafayette Avenue. Practice in surgery and gynaecology given special attention.

---

Dr. Hugh C. McDowell of Buffalo, in endeavoring to avoid running down a boy, turned his auto against the curb, was thrown out and badly injured as was the auto. Accidents of this sort, due to the carelessness of pedestrians are altogether too common. They show a general spirit of altruism that is commendable but often result in injury to more persons than if the person to blame suffered the logical consequences of his carelessness.

---

Dr. M. E. Leary of Rochester, Supt. of Iola Sanitarium, has been elected President of the State Sanitary Officers' Association, which he was instrumental in founding.

---

Dr. E. E. Blaauw of Buffalo has returned from Europe.

---

Dr. Milton P. Messinger of Oakfield, was at Saratoga Springs in September.

---

Dr. E. G. Truesdale of the Warsaw Board of Health is making a complete sanitary inspection of the buildings and premises, by authority of the State.

